# Prognostic nutritional index predicts clinical outcomes in patients with cerebral venous sinus thrombosis

**DOI:** 10.1186/s12883-021-02436-w

**Published:** 2021-10-21

**Authors:** Jiawei Zhao, Kai Liu, Shen Li, Yuan Gao, Lu Zhao, Hongbing Liu, Hui Fang, Jun Wu, Shilei Sun, Yusheng Li, Bo Song, Yuming Xu

**Affiliations:** Department of Neurology, the First Affiliated Hospital of Zhengzhou University, No.1 Eastern Jianshe Road, Zhengzhou, 450052 Henan China

**Keywords:** Prognostic nutritional index, Cerebral venous sinus thrombosis, Acute/subacute, Prognosis, Risk factors

## Abstract

**Background:**

Lower prognostic nutritional index (PNI) is related to the poor prognosis of cardiovascular diseases. However, little is known about PNI and its relationship with the prognosis of cerebral venous sinus thrombosis (CVST).

**Methods:**

CVST patients were retrospectively identified from January 2013 till June 2019. Patients in the acute / subacute phase were selected as subjects. Poor prognosis was defined as a modified Rankin Scale (mRS) of 3–6. Multivariate logistic regression analysis was used to confirm if lower PNI was associated with a poor prognosis.

**Results:**

A total of 297 subjects with follow-up data were enrolled. Thirty-three (11.1%) had a poor outcome. Multivariate logistic regression analysis suggested that PNI was an important predictive factor of poor outcome in acute/subacute CVST (odds ratio, 0.903; 95% CI, 0.833–0.978; *P* = 0.012). The optimal cut-off value for predicting the poor prognosis of PNI was 44.2. Kaplan-Meier analysis and log-rank test suggested that the lower the PNI value, the higher the mortality rate (*P* < 0.001). In addition, the nomogram that was set up showed that lower PNI was an index of poor prognosis. The c-index for acute/subacute patients with CVST was 0.872.

**Conclusion:**

Lower PNI is correlated with a higher risk of adverse clinical outcomes in patients with acute/subacute CVST.

**Supplementary Information:**

The online version contains supplementary material available at 10.1186/s12883-021-02436-w.

## Background

Cerebral venous sinus thrombosis (CVST) is a special type of cerebrovascular disease, which is characterized by cerebral venous reflux obstruction and intracranial hypertension caused by the disturbance of cerebrospinal fluid absorption. It is generally believed that the annual incidence of CVST is 2–5 cases / million, accounting for 0.5–1% of all strokes [1–6]. A recent report suggested a much higher incidence (13 cases / million every years) [7]. The inducing risk factors, clinical characteristics, and neuroimaging features of CVST are extremely diverse, which cause challenges to the prognosis [8]. Therefore, it is clinically significant to carry out early risk stratification at the time of onset.

Malnutrition has been proven to be an independent prognostic index of incidence and mortality in patients with various cancers, myocardial infarction (MI) as well as acute ischemic stroke (AIS) [9–11]. The PNI is a novel and comprehensive nutritional-inflammatory score that reflects the immunological nutritional status based on serum albumin concentration and lymphocyte count. Several studies have found that PNI is related to poor prognosis and increased mortality in patients with malignant diseases, MI and AIS [12–14]. Nevertheless, this correlation between PNI and prognosis in CVST patients has not been studied yet. Acute/subacute CVST is characterized by a sudden onset and rapid progression, which is of great significance to study [15]. Therefore, the aim of this study was to assess the outcome and predictive role of PNI in patients with acute/subacute CVST.

## Material and methods

### Patient selection

A follow-up study was conducted from January 2013 till June 2019 at the Henan CVST Registry in the First Affiliated Hospital of Zhengzhou University (Henan, China). The diagnosis of CVST complied with international standards [4, 5], based on clinical symptoms, patient history, and neuroimaging results (computed tomographic (CT), magnetic resonance venography (MRV) or digital subtraction angiography (DSA)). The acute period defined as the time from onset to hospital admission was less than 7 days. Whereas subacute period is defined as the time from onset to hospital admission was less than 30 days [16, 17]. The exclusion criteria as follows: (1) patients without complete clinical data; (2) time from onset to admission over 30 days [15]; (3) patients less than 18 years old; (4) patients with severe hepatic or renal diseases; (5) patients lost to follow-up. The registry study was approved by the Ethics Committee of the First Affiliated Hospital of Zhengzhou University.

### Data collection

Demographic data and information such as age, gender, risk factors, clinical manifestations, laboratorial parameters and imageological examination were gathered and analyzed. Albumin concentration (ALB) and the absolute lymphocyte count (ALC) were assessed 24 h after admission.

### Definition of PNI

Similar to previous studies, PNI was calculated using 5 ∗ lymphocyte count (10 ^9^ /L) + albumin concentration (g/L) [18].

### Outcome assessment

The outcome was assessed by modified Rankin Scale (mRS) during routine follow-up, which was performed over the phone after 3 months of discharge. The adverse prognosis was defined as mRS of 3–6. Telephone interviewers were unaware of baseline data.

### Statistical analysis

Statistical analysis was performed using SPSS 22.0. Continuous data were represented as mean ± SD, which were analyzed by independent Student’s t-test or Mann-Whitney U test, whichever was appropriate. Categorical variables were presented as numbers and percentages, which were compared by the χ^2^ test or Fisher exact test. Multivariate logistic regression analysis was used to analyze the association between PNI and clinical outcomes. The receiver operating characteristic (ROC) analysis was carried out to evaluate the ability of PNI in predicting clinical prognosis. The patients were dichotomized with the cut-off PNI value. The mortality rate in high and low PNI value groups was compared with the help of the log-rank test and plotted with the use of the Kaplan-Meier method. On this basis, a nomogram of independent predictive factors was established by using RMS software. The results were significant for Two-tailed *P* < 0.05.

## Results

### Study subject characteristics

Out of 324 patients who were diagnosed with CVST within 30 days of onset to admission and enrolled in the database, 27 patients were excluded: 15 patients younger than 18 years old, 8 patients without complete clinical data and 4 patients lost to follow-up. Therefore, 297 patients were included in the final analysis. The comparison of baseline characteristics and treatment details of included and excluded patients, showed no difference except for age (Table S1).

### Characteristics of patients with good and poor prognosis

During the follow-up period, 33 patients (11.1%) had poor functional outcomes, including 26 patients who died (8.8%). The baseline characteristics of the two groups are shown in Table 1. The PNI (49.3 ± 6.3 versus 42.3 ± 6.1, *P* < 0.001) of the poor outcome group considerably decreased. Patients with poor outcomes were older (34.2 ± 11.6 versus 44.4 ± 16.3; *P* < 0.001) compared with the good outcome group. In addition, coma, focal neurological deficits, involvement of straight sinus, and intracerebral hemorrhage in neuroimaging manifestations were more common in patients with adverse prognosis. The lymphocyte count and albumin concentration in subjects with adverse outcomes were lower than those with good outcomes (*P* < 0.001).

**Table 1:** Baseline characteristics according to the clinical functional outcome

	Good Outcome (*N* = 264)	Poor Outcome (*N* = 33)	*P*
Demographics			
Age, y, mean ± SD	34.2 ± 11.6	44.4 ± 16.3	< 0.001
Female, n (%)	155(58.7)	23(69.7)	0.225
Possible Risk factors, n (%)			
Infections	66(25.9)	8(24.2)	0.839
Pregnancy/postpartum	66(25.0)	7(21.2)	0.634
Clinical symptoms, n (%)			
Intracranial hypertension	175(66.3)	22(66.7)	0.965
Seizure	74(28.0)	12(36.4)	0.320
Coma	67(25.4)	23(69.7)	< 0.001
Mental status disturbance	13(4.9)	4(12.1)	0.200
Focal neurological deficits^a^	86(32.6)	18(54.5)	0.013
Involved sinuses, n (%)			
Transverse sinuses	99(37.5)	12(36.4)	0.899
Sigmoid sinuses	87(33.0)	9(27.3)	0.511
Superior sagittal sinus	106(40.2)	13(39.4)	0.933
Straight sinus	16(6.1)	6(18.2)	0.012
Inferior sagittal sinus	18(6.8)	3(9.1)	0.904
Deep CVT	9(3.4)	2(6.1)	0.786
Parenchymal lesion, n (%)			
Ischemic Infarction	40(15.2)	8(24.2)	0.181
Intracerebral hemorrhage	54(20.5)	15(45.5)	0.001
Laboratory Examinations			
White Blood Cell, 10^9^/L	9.6 ± 5.0	10.9 ± 5.5	0.165
Lymphocyte count, ×10^9^/L	1.6 ± 0.8	1.1 ± 0.5	< 0.001
Serum albumin, g/dL	40.5 ± 5.0	37.4 ± 5.0	0.001
PNI	49.3 ± 6.3	42.3 ± 6.1	< 0.001
Hospital treatment, n (%)			
Anticoagulation	262(99.2)	31(93.9)	0.062
Endovascular Therapies	125(47.3)	19(57.6)	0.268

*CVT* cerebral venous thrombosis; *PNI* prognostic nutritional index

^a^Focal neurological deficits symptoms included hemiplegia and sensory changes

### Analysis between PNI and long-term prognosis

**Fig. 1:**
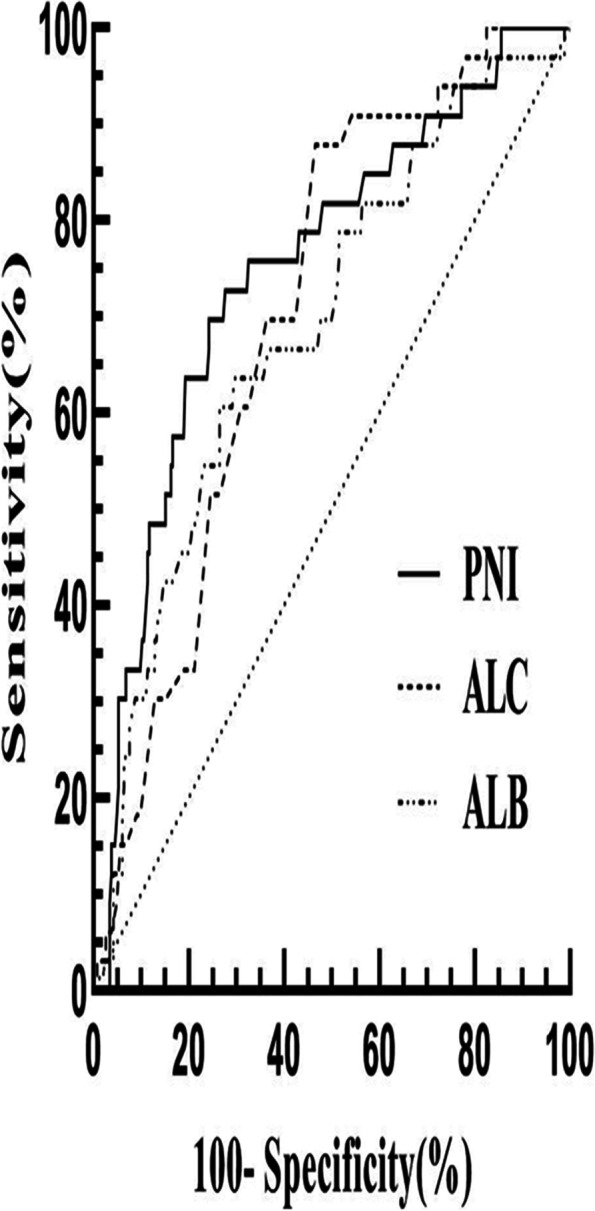
Receiver operating characteristic curves for the clinical functional outcome. Predictive values of ALC, ALB and PNI for the poor outcome. Area under the curve 0.71(95% CI, 0.63–0.79) for ALC, 0.69 (95% CI, 0.59–0.79) for ALB and 0.75(95% CI, 0.66–0.84) for PNI. ALC: absolute lymphocyte count, ALB: albumin concentration and PNI: prognostic nutritional index

**Fig. 2:**
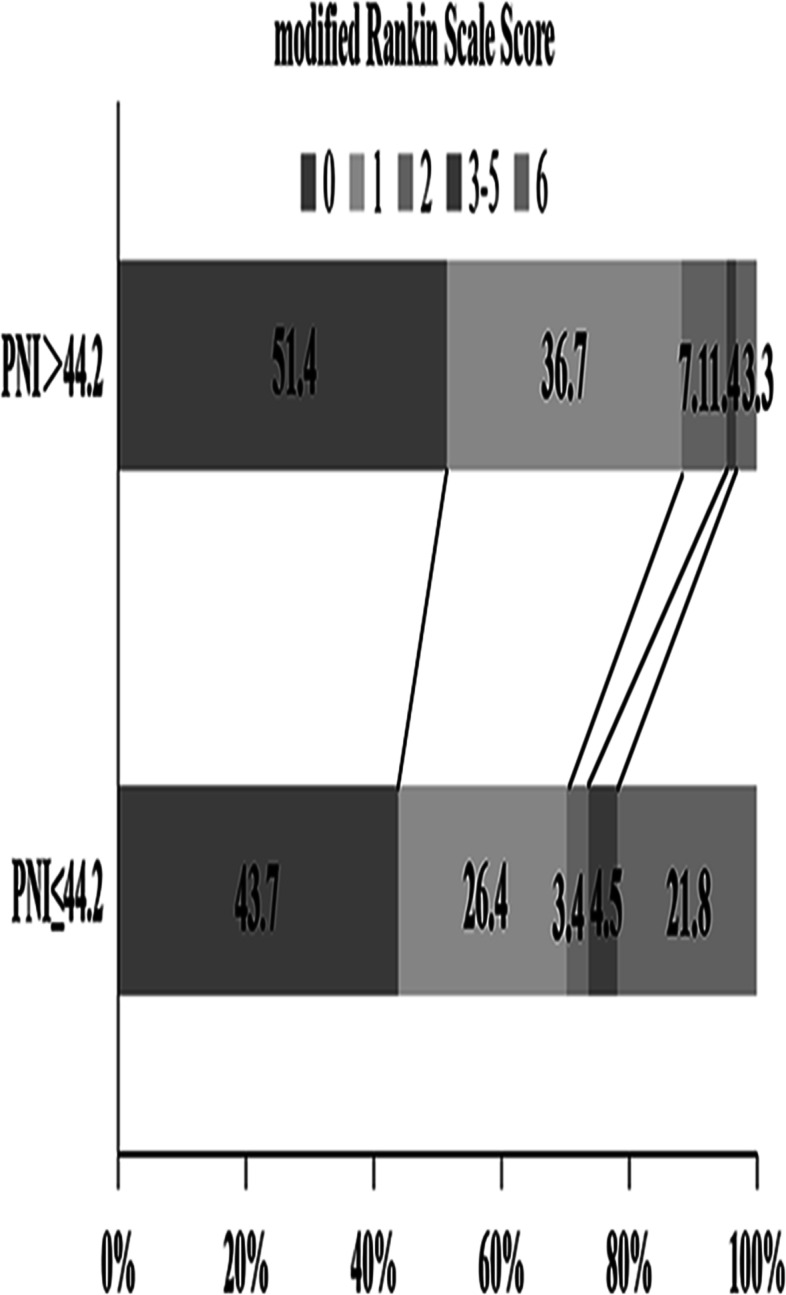
The mRS distribution for patients with PNI>44.2 and PNI ≤ 44.2 at the last follow-up. mRS, modified Rankin Scale; PNI: prognostic nutritional index

**Fig. 3:**
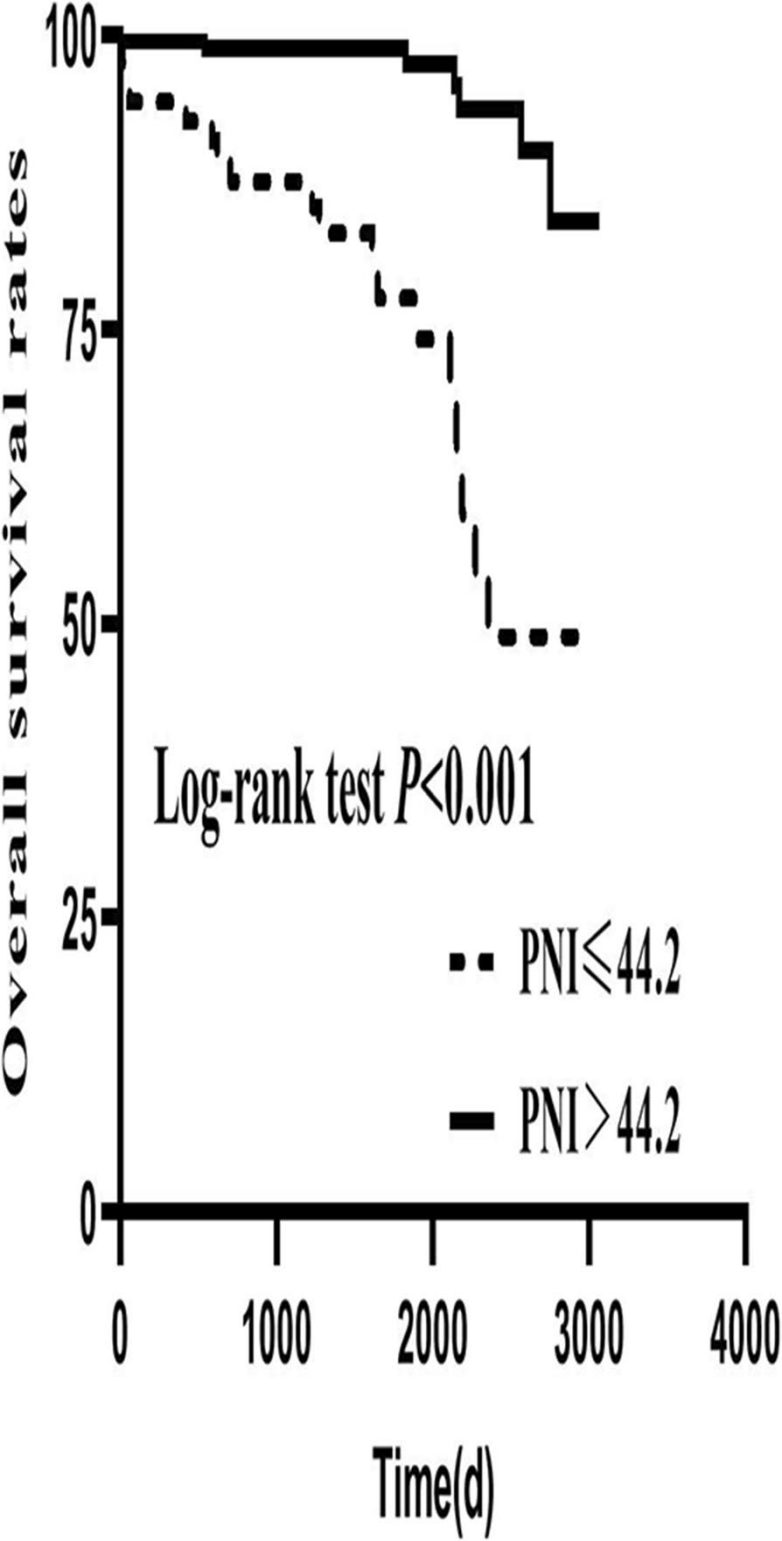
Kaplan-Meier curves of patients stratified according to the PNI value. The Kaplan-Meier curves showed a significant difference between the PNI>44.2 and PNI ≤ 44.2 categories (Log-rank test = 32.71, *P* < 0.001). PNI: prognostic nutritional index

As presented in Table 2, multivariate logistic regression analysis suggested that PNI (odds ratio, 0.903; 95% CI, 0.833–0.978; *P* = 0.012) remained significantly correlated with the functional outcome after the adjustment of potential confounders including age, gender, presence of coma, focal neurological deficits, intracerebral hemorrhage, and straight sinus. According to the ROC curve of laboratory parameters to predict poor results shown in Fig. 1, when 44.2 was set as the cut-off point, PNI showed the best predictive value with the receiver operating characteristic analysis and area under the curve (AUC) of 0.75 (sensitivity: 75.8% and specificity: 69.7%), which showed the highest AUC value than those of ALC (AUC 0.71; 95% CI, 0.63–0.79) and ALB (AUC 0.69; 95% CI, 0.59–0.79). It indicated that PNI provides a stronger predictive power of poor outcome than its constituent parts. The mRS distribution is shown in Fig. 2. Overall, patients with lower PNI had worse scores on the mRS than higher PNI patients. Kaplan-Meier analysis and log-rank test suggested that the lower the PNI value, the higher the mortality rate (*P* < 0.001) (Fig. 3).

**Table 2:** Multivariate logistic regression analysis of predictors for poor clinical outcome in CVST patients

Model	Variable	OR	95% CI	*P*
Model 1	Age	1.083	1.045 ~ 1.122	< 0.001
(with ALC)	Gender	2.102	0.787 ~ 5.615	0.138
	Coma	6.455	2.497 ~ 16.687	< 0.001
	Focal neurological deficits*	1.236	0.512 ~ 2.984	0.638
	Intracerebral hemorrhage	3.765	1.464 ~ 9.683	0.006
	Straight sinus	7.338	1.962 ~ 27.448	0.003
	ALC	0.420	0.189 ~ 0.936	0.034
Model 2	Age	1.081	1.044 ~ 1.119	< 0.001
(with ALB)	Gender	2.284	0.858 ~ 6.079	0.098
	Coma	6.941	2.692 ~ 17.899	< 0.001
	Focal neurological deficits*	1.273	0.527 ~ 3.079	0.592
	Intracerebral hemorrhage	3.585	1.408 ~ 9.128	0.007
	Straight sinus	9.006	2.272 ~ 35.695	0.002
	ALB	0.923	0.840 ~ 1.015	0.100
Model 3	Age	1.080	1.042 ~ 1.119	< 0.001
(with PNI)	Gender	2.094	0.777 ~ 5.641	0.144
	Coma	6.203	2.376 ~ 16.194	< 0.001
	Focal neurological deficits*	1.090	0.441 ~ 2.690	0.852
	Intracerebral hemorrhage	3.304	1.275 ~ 8.560	0.014
	Straight sinus	8.994	2.267 ~ 35.675	0.002
	PNI	0.903	0.833 ~ 0.978	0.012

*CVT* cerebral venous thrombosis; *ALC* absolute lymphocyte count; *ALB* albumin concentration; *PNI* prognostic nutritional index; *OR* odds ratio; *CI* confidence interval

### Nomograms to predict the prognosis

**Fig. 4:**
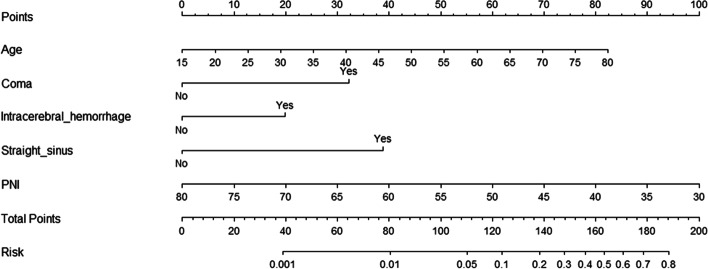
Nomograms of acute/subacute patients with CVST to predict the outcome

In addition, age, coma, intracranial hemorrhage, straight sinus involvement and PNI were included in the nomogram of the study (Fig. 4) by stepwise logistic regression analysis. The new model showed that older age, coma,  intracerebral hemorrhage, straight sinus, and lower PNI were indicators of poor prognosis. The c-index of the model was 0.872. These findings were similar to those of the previous multivariate logistic model.

## Discussion

In the International Study on Cerebral Vein and Dural Sinus Thrombosis (ISCVT), male gender, age > 37 years old, mental status disturbance, coma, thrombosis of the deep venous system, intracranial hemorrhage, malignancy, and infection of the central nervous system were independently associated with adverse outcomes at the last follow-up [19]. The VENOST study is the largest national multicenter study. Compared with previous studies, the clinical and imaging results are consistent; however, the susceptibility factors are different. The incidence of puerperium is high, and the use of oral contraceptives is not a common risk factor. The prognosis of malignant tumors, advanced age and hemorrhagic infarction is worse [20]. Our study showed that PNI was a significant and independent predictor of poor prognosis in non-chronic CVST patients.

The current research supports the link between inflammation and the incidence of CVST [21, 22]. A number of studies appeared to reinforce the biological plausibility behind inflammation and the prognosis of cerebral venous thrombosis [23, 24]. The lymphocyte count is an indicator that mediates cellular immunity. It is worth noting that some studies supported the use of lymphocyte count as a prognostic indicator. Lymphocytes are involved in the cellular immunity of various cancers and are related to cancer prognosis [25]. In addition, a previous study has shown that lymphocyte count is a predictive factor of adverse outcomes in patients with AIS [26]. In animal models, immunosuppression induced by stroke could lead to lymphopenia and changes in the ratio of helper T cells [27, 28].

Similarly, in human studies, it has also been observed that peripheral blood lymphocytes decreased after stroke, particularly in the acute phase [29, 30]. Therefore, lower lymphocyte count may be a predictor of adverse outcome. The relationship between lymphocyte count and the outcome of CVST has been confirmed in previous studies, such as platelet to lymphocyte ratio (PLR), lymphocyte to monocyte ratio (LMR) and neutrophil to lymphocyte ratio (NLR) [17, 31, 32].

Hypoalbuminemia is a comprehensive result of inflammation and insufficient intake of protein and calories in patients with chronic diseases. In different clinical settings, hypoalbuminemia has been shown to be a sign of poor prognosis [33–35]. Considering that albumin is a negative acute-- phase protein, its synthesis rate is affected by nutrition and inflammation [36]. More evidence shows that as the severity of inflammation increases, serum albumin levels gradually decrease [37, 38]. Albumin combined with nitric oxide (NO) free radicals has anticoagulant and antithrombotic effects. Due to the increase in the concentration of free lysophospholipids, hypoalbuminemia may affect blood viscosity and the function of endothelial cells [39]. However, serum albumin is not a good indicator of nutrition observation because it is greatly affected by fluid transfer [33]. Many different factors affect serum albumin levels and have shown a lack of sensitive and specific indicators of nutritional status [40].

As for PNI, it combines the lymphocyte count and albumin concentration, reflecting the nutrition, inflammation and immunity status. Therefore, compared with the factors mentioned above, PNI is more stable and representative. PNI was originally reported to be used to evaluate the immune and nutritional status of patients undergoing gastrointestinal tract surgery [18, 41, 42]. Subsequently, PNI has been widely used in prognostic evaluation of a variety of cancers and transplant operations, as well as for patients with various diseases such as myocardial infarction, acute type A aortic dissections and AIS patients receiving intravenous thrombolysis (IVT) [12, 13, 43, 44].

This study further showed that lower PNI increased the risk of poor outcomes in acute/subacute patients with CVST. Additionally, the findings from nomograms by stepwise logistic regression analyses suggested that age, coma, intracerebral hemorrhage, straight sinus involvement, and PNI were also predictors of adverse outcomes in acute/subacute patients with CVST, which further supported the results from multivariate logistic regression analyses. Therefore, the PNI created by combining serum albumin concentration and lymphocyte counts has the ability to assess the nutritional, inflammatory and immune status of patients with acute/subacute CVST. Considering that patients with lower PNI scores in this study have a significantly higher incidence of poor prognosis, it can be considered that appropriate evaluation and implementation of measures to improve nutritional status will help improve the outcome of patients with acute/subacute CVST. Further prospective studies are required to verify this hypothesis.

PNI is easy to obtain because it is calculated using objective laboratory test data. This makes it easy for our achievements to be translated into daily practice. However, several potential limitations should be acknowledged in the present study. Firstly, it is a single center study, and the sample size included in this study is relatively small. This association in the present study also needs to be confirmed and verified in larger multicenter prospective cohort studies. Secondly, compared with arterial stroke, it is difficult to determine the exact onset time in patients with CVST.

## Conclusion

In summary, our study suggested that lower PNI was a potential risk factor in unfavorable functional outcome of patients with acute/subacute CVST.

## Supplementary Information

**Additional file 1.**

## Data Availability

Study data are available from the corresponding author upon request.

## Abbreviations

CVST: Cerebral venous sinus thrombosis
PNI: Lower prognostic nutritional index
mRS: Modified Rankin Scale
MI: Myocardial infarction
AIS: Acute ischemic stroke
IVT: Intravenous thrombolysis
CT: Computed tomographic
MRV: Magnetic resonance venography
DSA: Digital subtraction angiography
ALB: Albumin concentration
ALC: Absolute lymphocyte count
ROC: Receiver operating characteristic
AUC: The receiver operating characteristic analysis and area under the curve
ISCVT: International Study on Cerebral Vein and Dural Sinus Thrombosis
PLR: Platelet to lymphocyte ratio
LMR: Lymphocyte to monocyte ratio
NLR: Neutrophil to lymphocyte ratio
NO: Nitric oxide
CVT: Cerebral venous thrombosis
OR: Odds ratio
CI: Confidence interval
