# Supplementary material for: Prognostic nutritional index predicts clinical outcomes in patients with cerebral venous sinus thrombosis
Source: BMC Neurol. 2021 Oct 21;21:404. doi: 10.1186/s12883-021-02436-w (PMC8529735; doi:10.1186/s12883-021-02436-w)
Supplement: Supplementary file 1 — Additional file 1. [file 12883_2021_2436_MOESM1_ESM.docx]

**Table S1 Comparison of the baseline characteristics between included and excluded patients**

|  | Excluded patients  (N=27) | Included patients  (N=297) | *P* |
| --- | --- | --- | --- |
| Demographics |  |  |  |
| Age, y, mean ± SD | 21.9±11.9 | 35.3±12.6 | <0.001 |
| Female, n (%) | 12(44.4) | 178(59.9) | 0.118 |
| Possible Risk factors, n (%) |  |  |  |
| Infections, n (%) | 7(28.0) | 74(25.7) | 0.801 |
| Pregnancy/postpartum | 3(11.1) | 73(24.6) | 0.114 |
| Clinical symptoms, n (%) |  |  |  |
| Intracranial hypertension | 19(70.4) | 197(66.3) | 0.670 |
| Seizure | 6(22.2) | 86(29.0) | 0.458 |
| Coma | 7(25.9) | 90(30.3) | 0.634 |
| Focal neurological deficits* | 5(18.5) | 104(35.0) | 0.082 |
| Involved sinuses, n (%) |  |  |  |
| Transverse sinuses | 14(51.9) | 111(37.4) | 0.139 |
| Sigmoid sinuses | 10(37.0) | 96(32.3) | 0.617 |
| Superior sagittal sinus | 13(48.1) | 119(40.1) | 0.413 |
| Straight sinus | 3(11.1) | 22(7.4) | 0.754 |
| Inferior sagittal sinus | 2(7.4) | 21(7.1) | 0.948 |
| Parenchymal lesion, n (%) |  |  |  |
| Ischemic Stroke | 3(11.1) | 48(16.2) | 0.679 |
| Intracerebral hemorrhage | 5(18.5) | 69(22.6) | 0.629 |
| Hospital treatment, n (%) |  |  |  |
| Anticoagulation | 25(92.6) | 279(93.9) | 0.781 |
| Endovascular Therapies | 10(37.0) | 144(48.5) | 0.254 |
